# IL-10-Producing Th1 Cells and Disease Progression Are Regulated by Distinct CD11c^+^ Cell Populations during Visceral Leishmaniasis

**DOI:** 10.1371/journal.ppat.1002827

**Published:** 2012-07-26

**Authors:** Benjamin M. J. Owens, Lynette Beattie, John W. J. Moore, Najmeeyah Brown, Jason L. Mann, Jane E. Dalton, Asher Maroof, Paul M. Kaye

**Affiliations:** Centre for Immunology & Infection, Hull York Medical School and Department of Biology, University of York, York, United Kingdom; National Institute for Medical Research, United Kingdom

## Abstract

IL-10 is a critical regulatory cytokine involved in the pathogenesis of visceral leishmaniasis caused by *Leishmania donovani* and clinical and experimental data indicate that disease progression is associated with expanded numbers of CD4^+^ IFNγ^+^ T cells committed to IL-10 production. Here, combining conditional cell-specific depletion with adoptive transfer, we demonstrate that only conventional CD11c^hi^ DCs that produce both IL-10 and IL-27 are capable of inducing IL-10-producing Th1 cells *in vivo*. In contrast, CD11c^hi^ as well as CD11c^int/lo^ cells isolated from infected mice were capable of reversing the host protective effect of diphtheria toxin-mediated CD11c^+^ cell depletion. This was reflected by increased splenomegaly, inhibition of NO production and increased parasite burden. Thus during chronic infection, multiple CD11c^+^ cell populations can actively suppress host resistance and enhance immunopathology, through mechanisms that do not necessarily involve IL-10-producing Th1 cells.

## Author Summary

Dendritic cells are well known as myeloid cells that bridge innate and adaptive immunity, and play an important role in the induction of cell-mediated immunity to a variety of pathogens. However, very little is known about the function of dendritic cells after infection has become established. In this study, we have examined the role of dendritic cells during the later phases of experimental visceral leishmaniasis, caused by infection with *Leishmania donovani*. We show for the first time that dendritic cells are responsible for promoting the development of splenic pathology, and that their removal during established infection leads to improved host resistance. Furthermore, our studies provide the first formal evidence *in vivo* that dendritic cells making IL-27 can induce production of the regulatory cytokine IL-10 by effector Th1-like CD4^+^ T cells. Surprisingly, we also found that other populations of CD11c^+^ cells were able to induce pathology and suppress host resistance, yet did not stimulate IL-10 production in CD4^+^ T cells, suggesting that the latter T cell population may not play an essential role in disease progression. Our studies provide new insights into dendritic cell function in chronic parasite infection and suggest potential new avenues for immunotherapy against visceral leishmaniasis.

## Introduction

Dendritic cells (DCs) are widely recognized as being the most important myeloid cell involved in antigen presentation and the initiation and regulation of CD4^+^ T cell-dependent protective immunity against a variety of intracellular parasites (reviewed in [1], [2]), and show promise for the development of new approaches in vaccination and immunotherapy [3], [4]. Initially based largely on *in vitro* studies, the key role of DCs in antigen presentation has been borne out in recent years through the availability of mice in which DCs can be ablated in a conditional manner [5]. Hence, diphtheria toxin (DTx)-mediated ablation of DCs results in a significant reduction in T cell priming following various infectious challenges, including with *Mycobacterium tuberculosis*, *Plasmodium yoelli*, *Listeria monocytogenes*, *Streptococcus pyogenes* and LCMV [6], [7], [8], [9]. In contrast, the role of DCs during later stages of infection and their contribution to the immune imbalance that is often associated with chronic infection are less well understood, in spite of the known ability of DCs to induce tolerogenic or regulatory responses [4], [10], [11], [12].

CD11c^+^ DCs play multiple roles in the pathogenesis of leishmaniasis, including experimental visceral leishmaniasis (EVL) caused by *Leishmania donovani* (reviewed in [13]). Dermal DC [14] and Langerhans cells [15] have been implicated in the early stages of *L. major* infection, and as this infection progresses, many parasites are found in the draining LN within CD11c^+^ cells that resemble TipDCs [16]. Expression of MHCII on DCs is both necessary and sufficient for the induction of effective immunity to *L. major*, suggesting macrophage antigen presentation may not be required for effector T cell function [17]. In EVL, splenic DCs belonging to the CD8α subset of conventional DCs (cDCs) are the first cells to produce IL-12 within the splenic microenvironment [18], and are activated for heightened expression of a variety of costimulatory molecules through both direct interactions with *Leishmania* parasites and through inflammatory signals [19]. In chronic EVL, however, cDC cytokine production is modulated in a subset-specific manner [18] and migration through lymphoid tissue is disrupted [20]. In addition, CD11c expression is found on other cells known to contribute to anti-leishmanial resistance, including NK cells [21], and inflammatory monocytes/TipDCs [16]. However, the relative contribution of these different CD11c^+^ cell populations to disease progression and the regulation of T cell effector and regulatory function is poorly understood.

Visceral leishmaniasis is also noted for the production of the immunoregulatory cytokine IL-10, and targeting of IL-10 signaling has been identified as a potential therapeutic strategy [22]. Although multiple cellular sources of IL-10 have been identified in VL, the identification of a population of IFNγ-producing CD4^+^ T cells that also produces IL-10 and its association with progressive disease in both mice [23], [24] and in humans [25] has drawn particular attention. The co-production of IL-10 by IFNγ-producing CD4^+^ T cells is not novel for leishmaniasis, however, and is now a recognized feature of Th1 cell differentiation. Considerable attention has been focused, therefore, on dissecting the molecular signals required for expression of this mixed effector/regulatory phenotype. *In vitro* studies using transgenic CD4^+^ T cells and repeated exposure to antigen and APCs have suggested that the induction of IL-10 is a consequence of sustained antigen presentation, requiring the presence of high levels of IL-12 [26]. The cytokine IL-27 is also implicated in the generation of IL-10-producing CD4^+^ T cells *in vitro* [27], [28], [29], [30], [31], via signaling pathways dependent on STAT3 [29], and optimal generation of CD4^+^IL-10^+^ cells by IL-27 requires the co-ordinate initiation of c-Maf, ICOS and IL-21 expression [32], [33]. In addition, emerging evidence suggests that IL-27 may directly alter methylation patterns around the *il10* promoter in CD4^+^ T cells, thus allowing greater IL-10 expression [34]. IL-27 also favors the production of IL-10 by IFNγ-producing Th1 cells through an alternate signaling pathway that involves STAT1, STAT4 and Notch [35], [36]. In spite of these advances, the cellular sources of IL-27 *in vivo* have been poorly defined. A direct role for DC-derived IL-27 in the generation of IL-10^+^ T cells has been described *in vitro*, where production of this cytokine in response to galectin-1, and during ovalbumin-induced oral tolerance, both favored the differentiation of IL-10-producing T cells with potent regulatory capacity [37], [38]. Nevertheless, the cellular requirements for generating CD4^+^IFNγ^+^IL-10^+^ T cells *in vivo* remain obscure and no studies to date have addressed this question in the context of chronic infection.

We therefore sought to address two inter-related questions: i) what is the role of CD11c^+^ cells during chronic *L. donovani* infection and ii) do these cells contribute to the emergence of IL-10-producing Th1 cells. Given recent concerns over off target effects induced by DTx treatment of mice [8], [39], we used a functional complementation approach to independently examine the role of CD11c^hi^ cDCs and CD11c^int/lo^ cells in determining host resistance and Th1 cytokine production. We show that CD11c^hi^ cDCs, as well as a mixed CD11c^int/lo^ cell population, are capable of inhibiting host resistance and promoting disease-associated pathology. Our study also provides the first formal evidence that IL-10^+^IL-27^+^ cDCs are able to promote IL-10 production by Th1 cells *in vivo*. Our data therefore highlight CD11c^+^ cells as potential targets for immunotherapy and also demonstrate an important discordance between disease progression and the emergence of IL-10-producing Th1 cells.

## Results

### IFNγ^+^IL-10^+^ T-bet^+^ CD4^+^ T cells expand during chronic *L. donovani* infection

**Figure 1:**
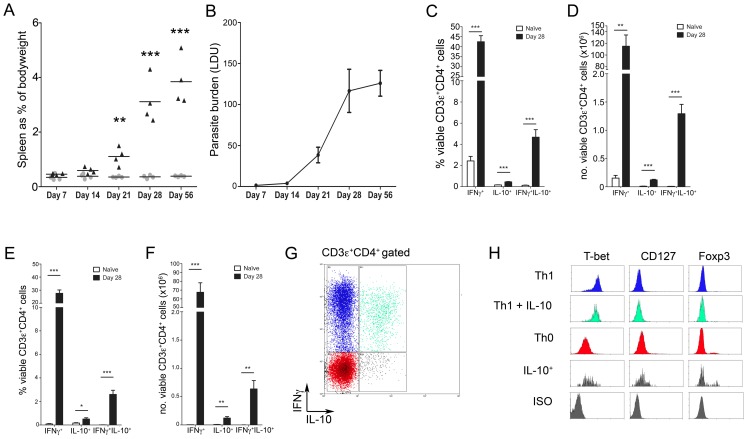
IFNγ^+^IL-10^+^ T-bet^+^ CD4^+^ T cells expand during chronic *L. donovani* infection. (**A and B**) C57BL/6 mice were infected i.v. with 3×10^7^ *Leishmania donovani* amastigotes, and at the indicated times post infection (p.i.), splenomegaly (**A**) and tissue parasite burden (**B**) were determined. (**C and D**) Frequency (C) and total number (D) of IL-10 and IFNγ producing splenocytes in naïve and day 28 infected mice after PMA and Ionomycin stimulation. (**E and F**) Frequency (E) and total number (F) of IL-10 and IFNγ producing splenocytes in naïve and day 28 infected mice after antigen stimulation. (**G and H**) T-bet, CD127 and Foxp3 expression (H) by different cytokine producing populations of CD3ε^+^CD4^+^ T cells from d28 infected mice (G). Data are from n = 4 mice per group and representative of 2–3 independent experiments. * = p<0.05, ** = p<0.01, *** = p<0.001 for infected vs. naïve mice.

C57BL/6 mice infected with *L. donovani* amastigotes developed pronounced splenomegaly from day 21 post infection (p.i.) (**Figure 1A**), associated with an increasing tissue parasite burden (**Figure 1B**). As assessed by polyclonal activation *ex vivo* (**Figure 1C and D**), the frequency of splenic CD3ε^+^CD4^+^ IFNγ^+^ T cells increased from 2.4±0.4% in naïve mice to 44.4±3.0% (p<0.001) by day 28 of infection which, when taking into account splenomegaly, reflected a >500-fold increase in absolute number of cells committed to IFNγ production in the spleen. Infection was also associated with emergence of a population of splenic CD3ε^+^CD4^+^ T cells capable of producing both IFNγ and IL-10. This population increased in frequency ∼50 fold, comprising 0.1±0.02% of CD3ε^+^CD4^+^ cells in naïve mice and 5.0±0.9% at day 28 of infection (p<0.001), equating to a ∼170 fold expansion in the total number of splenic T cells capable of simultaneous production of IFNγ and IL-10 (7.6±1.2×10^3^ vs 1.3×10^6^±1.7×10^5^ in naïve and day 28 infected mice, respectively; p<0.001). Whilst the frequency and number of CD3ε^+^CD4^+^ T cells producing IL-10 alone also increased, this population of IL-10-producing cells remained modest compared to those that also made IFNγ. Similar results were also obtained following *in vitro* re-stimulation of splenic CD4^+^ T cells using *Leishmania*-antigen pulsed BMDCs (**Figure 1E and F**).

In order to determine the lineage origin of these IFNγ^+^IL-10^+^ CD4^+^ T cells, cytokine producing cells (**Figure 1G**) were examined for intracellular expression of the Th1-associated transcription factor *Tbx21* (T-bet), the regulatory T cell-associated transcription factor forkhead box transcription factor 3 (Foxp3), and for surface expression of the IL-7 receptor alpha chain (CD127). CD3ε^+^CD4^+^ T cells capable of simultaneous production of IFNγ and IL-10 were exclusively T-bet^+^, CD127^−^ and Foxp3^−^ (**Figure 1H**). In contrast to the expansion of IL-10-producing CD4^+^ T cells, the frequency of splenic Foxp3^+^ T_reg_ did not increase during infection (**Figure S1**), but instead decreased as previously reported [23]. Therefore, chronic infection with *L. donovani* was associated with the expansion of antigen-specific CD4^+^ T-bet^+^CD127^−^ Foxp3^−^ ‘Th1-like’ cells capable of simultaneously producing IFNγ and IL-10.

### CD11c^hi^ splenic cDCs acquire a regulatory profile

**Figure 2:**
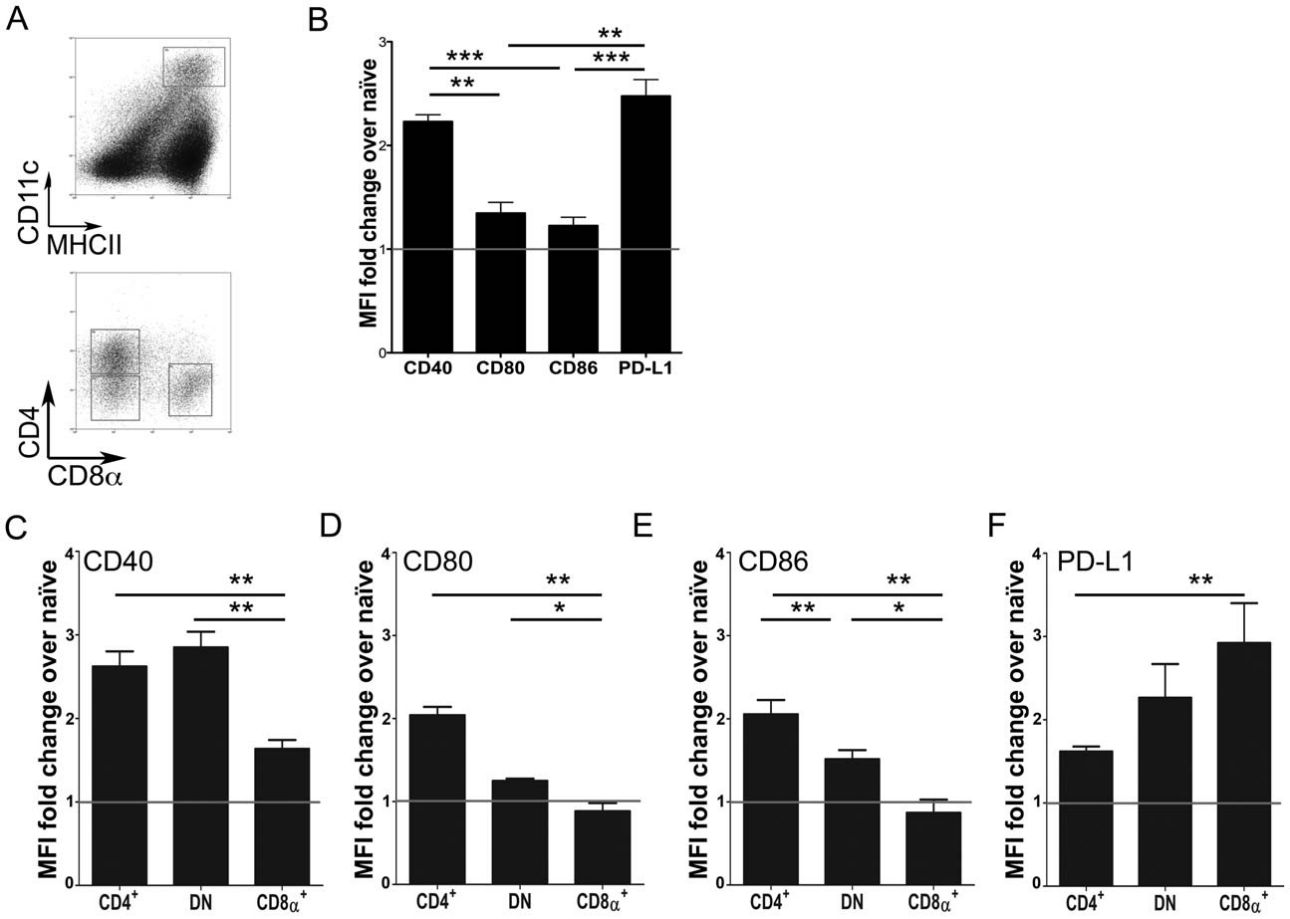
Costimulatory molecule expression on cDCs during chronic infection. (**A**) Splenic cDCs were gated as MHCII^hi^CD11c^hi^ cells (top panel) and further gated into the three major subsets based on CD4 and CD8α expression (bottom panel). (**B**) Surface expression of CD40, CD80, CD86 and PD-L1 on the entire cDC population. (**C–F**) Surface expression of CD40 (C), CD80 (D), CD86 (E) and PD-L1 (F) by each cDC subset. Data are expressed as mean fold change ± SEM of the MFI on cDCs from infected mice compared to naïve mice (n = 4 mice per group). Data is representative of 3 experiments.

To address the cellular mechanisms underlying the expansion of CD4^+^IFNγ^+^IL-10^+^ T cells, we focused on alterations within the splenic CD11c^hi^MHCII^hi^ cDC compartment (**Figure 2A**). First, we characterized the entire cDC population for the expression of cell surface ligands associated with costimulation. CD80 and CD86 expression was not changed compared to that on cDCs from naïve mice and only a 2–2.5 fold induction of CD40 and PD-L1 expression was noted on cDCs at day 28 p.i., relative to naïve mice (**Figure 2B** and **Figure S2**). The three major lymphoid-resident cDC subsets, as determined by CD4 and CD8 expression (**Figure 2A**), were all found at the expected frequencies [40], but individual subsets showed some level of differential regulation of co-stimulatory molecule expression during infection (**Figure 2C–F**). Of note, CD8α^+^ cDCs showed the least activation as judged by CD40, CD80 and CD86 expression, yet conversely had the greatest increase in PD-L1 expression.

**Figure 3:**
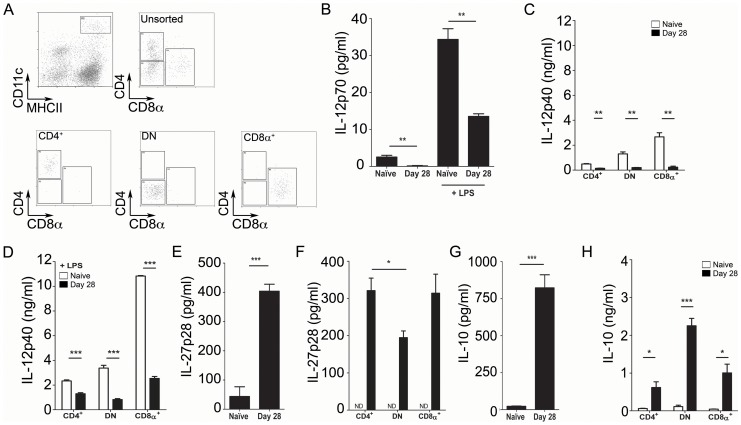
CD11c^hi^ splenic cDCs acquire a regulatory cytokine profile. (**A**) Sorting strategy and representative sort purities of unfractionated cDCs (top left panel) and each sorted cDC subset. (**B**) IL-12p70 secretion by CD11c^hi^MHCII^hi^ cDCs from naïve and/or day 28 infected mice, in the presence or absence of LPS. (**C and D**) Secretion of IL-12/23p40 by cDC subsets in the absence (B) or presence (C) of LPS. (**E and F**) Spontaneous IL-27p28 secretion by unfractionated cDCs (E) and sorted cDC subsets (F). (**G and H**) IL-10 secretion by unfractionated cDCs (G) and sorted (H) cDC subsets.

In light of the critical role for APC-derived cytokines in shaping CD4^+^ T cell lineage commitment, we next sought to determine how chronic infection impacted upon cDC cytokine production *ex vivo*. Highly purified CD11c^hi^MHCII^hi^ cDCs from spleens of naïve and day 28-infected mice (**Figure 3A**) showed differential cytokine profiles at both the whole population level and when separated into distinct subsets. CD11c^hi^MHCII^hi^ cDCs from day 28 infected mice had significantly reduced levels of spontaneous and LPS-induced IL-12p70 secretion, when compared to cDCs from naïve mice (p<0.01; **Figure 3B**). Similar results were also obtained from isolated cDC subsets by analysis of IL-12p70 [18] and IL-12/23p40 secretion (**Figure 3C and D**). In contrast, cDC production of IL-27p28 was significantly enhanced during infection, both at the bulk population level (**Figure 3E**) and when assessed for each individual cDC subset (**Figure 3F**). Similarly, cDCs from infected mice also produced elevated levels of IL-10 when compared to cDCs from naïve mice (**Figure 3G and H**). Although more pronounced by day 28 p.i., similarly altered cytokine responses have been observed in the early stages of acute *L. donovani* infection [18].

**Figure 4:**
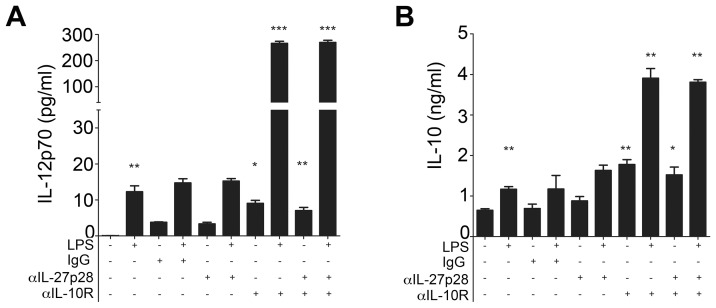
Autocrine IL-10 but not IL-27p28 regulates IL-12 and IL-10 production by cDCs. (A) IL-12p70 and (B) IL-10 secretion was determined in cultures of cDCs sorted from d28 infected mice cultured with or without LPS and in the presence or absence of neutralizing mAbs to IL-10R or IL-27p28. Data are representative of 3 experiments. * = p<0.05, ** = p<0.01, *** = p<0.001.

Autocrine IL-10 signaling is known to influence DC cytokine production, with splenic cDCs particularly sensitive to this form of regulation [41], [42]. Therefore, we next sought to determine whether the altered cDC cytokine profile was in part dependent on autocrine IL-10 and/or IL-27 production. cDCs from infected mice cultured in the presence of αIL-10R mAb spontaneously secreted IL-12p70 to a similar extent to those treated with LPS (**Figure 4A**). However, maximal IL-12p70 production was achieved by simultaneous IL-10R blockade and LPS stimulation. IL-10R blockade also significantly enhanced the accumulation of IL-10 in the culture medium, again most pronounced when combined with LPS stimulation (**Figure 4B**). These data indicate a potent negative regulatory function for autocrine IL-10 produced by cDCs isolated from infected mice. In contrast, neutralization of IL-27p28 alone had no impact on *ex vivo* IL-12p70 or IL-10 production by cDCs isolated from infected mice, nor was there any additive effect when combined with IL-10R blockade (**Figure 4A and B**). Hence, IL-27p28 does not auto-regulate cDC cytokine production under these conditions.

Finally, we examined whether the immunomodulators TGFβ or Indoleamine 2,3-dioxygense (IDO) were substantially regulated in cDC subsets as a result of infection with *L. donovani*. At the transcriptional level, only CD4^+^ cDCs from infected mice showed any significant increase in accumulation of *Tgfβ* mRNA (**Figure S3A**) and in no population of cDC did we observe any accumulation of *Ido* mRNA as a result of infection (**Figure S3B**). In summary, therefore, chronic *L. donovani* infection is associated with muted co-stimulatory molecule expression, increased IL-10 and IL-27p28 production and a dramatic impairment in IL-12p70 production by splenic cDCs, with IL-12p70 secretion regulated in part by autocrine IL-10 signaling.

### Therapeutic depletion of CD11c^+^ cells enhances host resistance and reduces disease pathology

**Figure 5:**
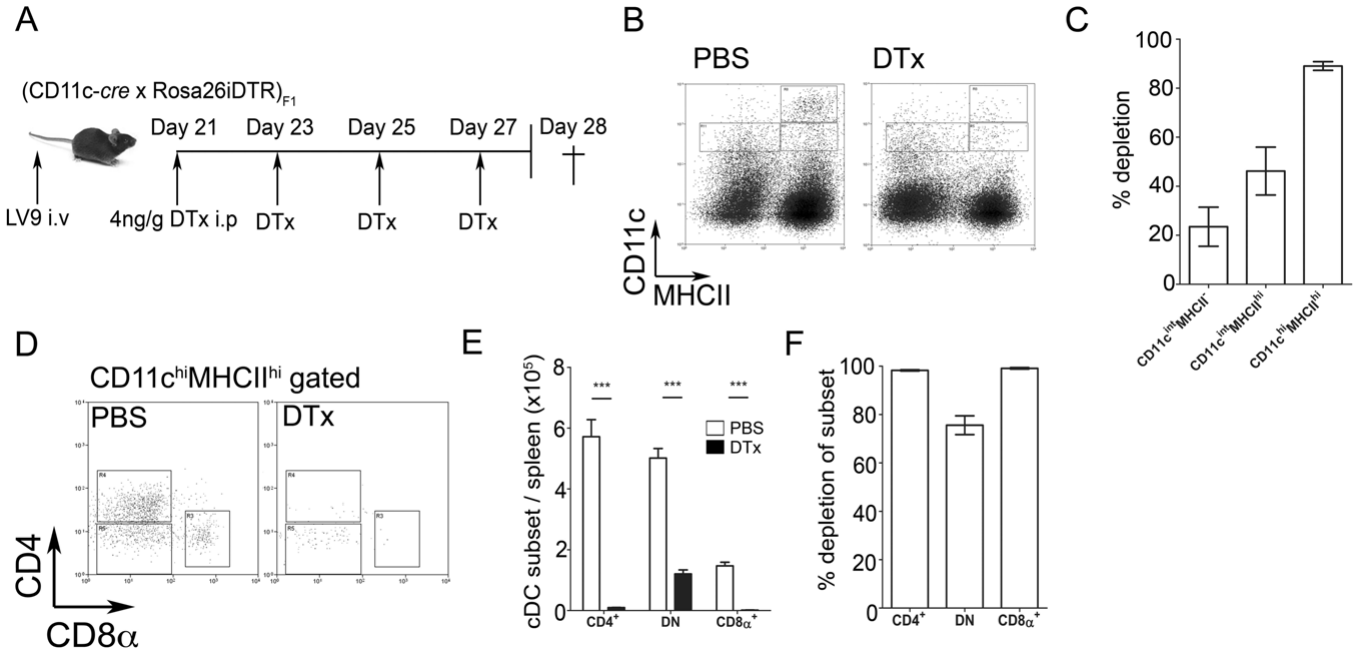
Deletion of CD11c^+^ cells in DTx treated (CD11c-*cre*×Rosa26iDTR)_F1_ mice. (**A**) Experimental protocol: Infected (CD11c-*cre*×Rosa26iDTR)_F1_ mice received 4 ng/g DTx i.p. at 48 hour intervals from d21 until day 28 p.i. (**B and C**) Impact of CD11c^+^ cell ablation on cells expressing CD11c and MHCII, shown by representative dot plot (B) and by extent of depletion CD11c^lo^MHCII^−^, CD11c^lo^MHCII^hi^ and CD11c^hi^MHCII^hi^ (cDCs) relative to untreated mice (C). (**D–F**) Impact of DTx treatment on CD11c^hi^ cDC subsets, shown by representative dot plot (D), as total number of cells in DTx treated (black bars) vs. control (open bars) mice (E) and by extent of depletion (F). Data are from 2 pooled experiments (n = 8 mice). *** = p<0.001 for DTx vs. PBS treated groups.

To assess the *in vivo* impact of cDCs during chronic infection, we generated (CD11c-*cre*×Rosa26iDTR)_F1_ mice. In these mice, expression of *Cre* recombinase is driven by CD11c promoter activity, resulting in cleavage at *loxP* sites flanking a ubiquitously expressed STOP cassette upstream of a simian diphtheria toxin receptor (DTR). Past or current expression of CD11c initiates DTR expression and thus provides inducible sensitivity to diphtheria toxin (DTx). We administered either saline or DTx i.p. to (CD11c-*cre*×Rosa26iDTR)_F1_ mice at 48 hour intervals for a period of 7 days, beginning on day 21 p.i. (**Figure 5A**). Unlike in CD11c-DTR mice [5], we found no evidence of toxicity using this regimen. ∼80–90% of CD11c^hi^MHCII^hi^ cDCs were ablated after 7 days of treatment (**Figure 5B and C**). Depletion was almost 100% complete for the CD4^+^ and CD8α^+^ subsets, with most residual cDCs belonging to the DN subset (**Figure 5D–F**). In addition to depletion of cDCs, we also observed depletion of some CD11c^int/lo^ cells (**Figure 5B and C**). ∼20% of CD11c^int/lo^MHCII^−^ cells and 40% of CD11c^int/lo^MHCII^hi^ cells were lost, most likely including both CD11c^int/lo^ DCs and NK cells [21], [43]. Additional off target effects of DTx treatment were also noted. CD169^+^ marginal zone macrophages were depleted, as determined by immunofluorescence staining of tissue sections (data not shown). Although others have observed loss of marginal zone macrophages in CD11c-DTR mice [39], these cells are already largely absent in mice infected with *L. donovani* [44]. Other significant off-target effects of DTx treatment were restricted to a decrease in the frequency and number of NK1.1^+^CD11b^+^ NK cells and an increase in the frequency of splenic CD11b^hi^Gr-1^hi^ neutrophils (1.46±0.18% vs. 2.38±0.29% in infected vs. DTx-treated infected mice respectively; p<0.05, **Figure S4**).

**Figure 6:**
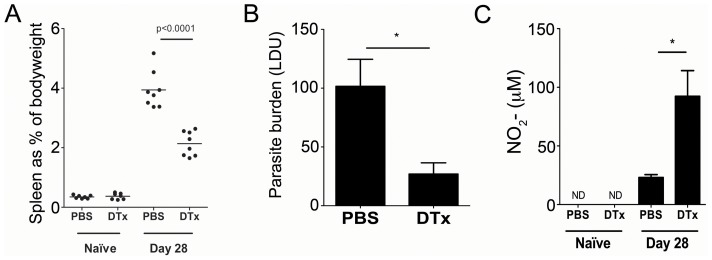
Deletion of CD11c^+^ cells reduces pathology and enhances resistance to *L. donovani*. Infected (CD11c-*cre*×Rosa26iDTR)_F1_ mice were treated as shown in Figure 6A and at d28 p.i. mice were assessed for splenomegaly (**A**), spleen parasite burden (**B**) and spontaneous NO production by adherent splenocytes (**C**). Data are pooled from two independent experiments (n = 8 mice). * = p<0.05, other p values as indicated, for DTx vs. PBS treated animals.

Depletion of CD11c^+^ cells in chronically infected (CD11c-*cre*×Rosa26iDTR)_F1_ mice had a profound impact on splenic pathology, reflected by a dramatically reduced spleen size (from 3.94±0.22% vs. 2.14±0.14% of total body weight in saline-treated and DTx-treated mice, respectively; p<0.0001). In contrast, treatment of naïve (CD11c-*cre*×Rosa26iDTR)_F1_ mice with DTx had no impact on spleen size (**Figure 6A**). Ablation of CD11c^+^ cells also significantly reduced splenic parasite burden (101±23 vs. 27±9 LDU in saline vs. DTx treated mice, respectively; p<0.05; **Figure 6B**). Conversely, mice treated with DTx had an almost 5-fold increase in nitric oxide production, as measured by spontaneous release from adherent splenocytes (23.04±2.51 vs. 92.36±21.89 µM control and DTx treated mice; p<0.05). NO production was not detectable from adherent splenocytes in naïve animals, irrespective of DTx treatment (**Figure 6C**). By each of these criteria, therefore, CD11c^+^ cells appeared to be playing a role in promoting disease progression *in vivo*.

### Therapeutic depletion of CD11c^+^ cells inhibits the development of IL-10-producing Th1 cells

**Figure 7:**
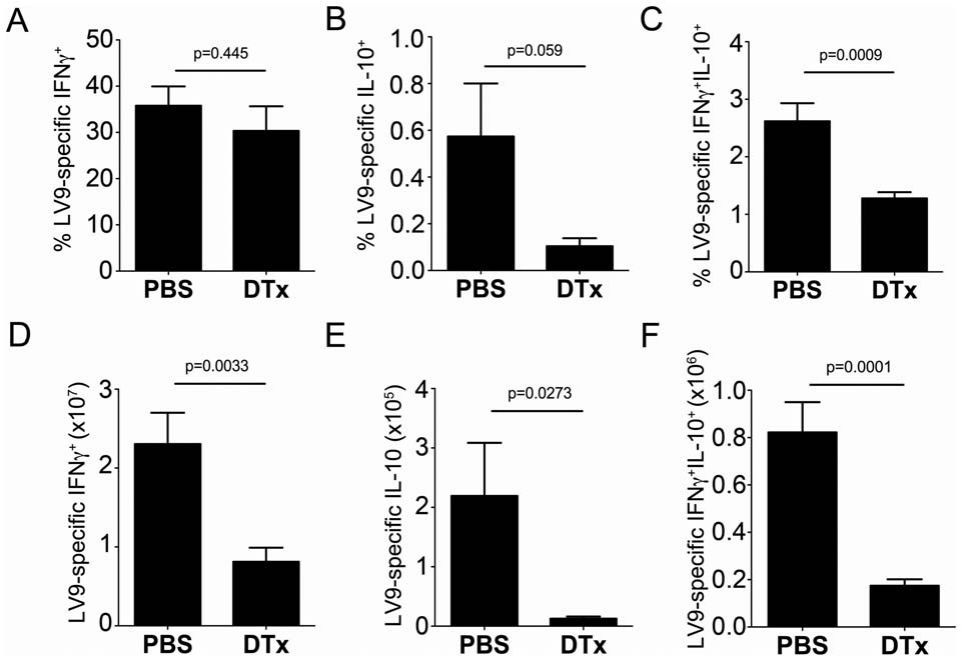
CD11c^+^ cells regulate IL-10 production by Th1 cells. Splenic T cells from the mice used in Figure 7 were analysed for cytokine production (**A**–**C**) Frequency of antigen-specific CD4^+^ T cells producing IFNγ (A), IL-10 (B) or both (C) in mice with or without DTx treatment. (**D**–**F**) Total number of antigen-specific CD4^+^ T cells producing IFNγ (D), IL-10 (E) or both (F) in mice with and without DTx treatment. Data are pooled from two independent experiments (n = 8 mice). * = p<0.05, other p values as indicated, for DTx vs. PBS treated animals.

The experimental approach outlined above provided an opportunity to determine whether there was a causal link between the appearance of the phenotypically distinct cDCs described above and the induction of IL-10-producing Th1 cells. We therefore examined CD4^+^ T cells from these DTx-treated mice for their capacity to produce IFNγ and IL-10 (**Figure 7**). Depletion of CD11c^+^ cells from day 21 to day 28 of infection did not affect the frequency of antigen-specific IFNγ^+^ T cells (**Figure 7A**; 35.78±4.18% vs. 30.33±5.32% after saline or DTx, respectively), although absolute numbers were decreased by approximately 2-fold in keeping with the reduction of spleen size (**Figure 7D**). In contrast, the frequency of antigen-specific CD4^+^ T cells capable of the simultaneous production of IFNγ and IL-10 was significantly reduced following DTx administration (**Figure 7C**; 2.62±0.31% vs. 1.28±0.11% in saline and DTx treated mice respectively; p<0.001) and the absolute number per spleen was reduced by four fold (**Figure 7F**). Antigen-specific CD3ε^+^CD4^+^ T cells capable only of IL-10 production also showed a trend towards a reduction in frequency, but this was not significant (0.57±0.23% to 0.10±0.03%; p = ns; **Figure 7B**). The absolute number of these cells in the spleen was significantly reduced, however (**Figure 7E**). In combination, these data demonstrate that depletion of CD11c^+^ cells during chronic infection dramatically reduces splenic pathology, allows NO-dependent parasite clearance and impairs the generation of antigen-specific CD3ε^+^CD4^+^ T cells capable of simultaneous IL-10 and IFNγ production.

**Figure 8:**
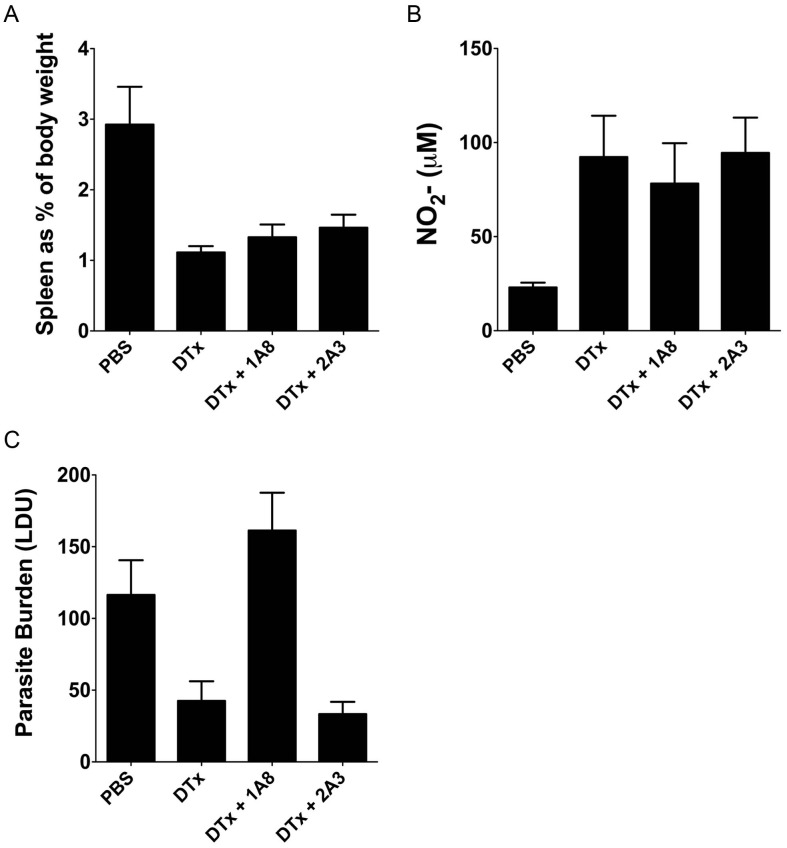
Removal of CD11c^+^ cells affects splenomegaly and NO production even in the absence of neutrophils. DTx-treated (CD11c-*cre*×Rosa26iDTR)_F1_ mice were administered neutrophil depleting mAb 1A8 or control mAb (2A3) every 48 h from d21 post infection. Mice were analyzed at day 28 post infection. (**A–C**) Splenomegaly and NO production (B) and spleen parasite burden (C) at d28 post infection in neutrophil depleted and neutrophil replete DTx-treated mice. Data are shown as mean ± SEM (n = 3 or 4 mice per group).

**Figure 9:**
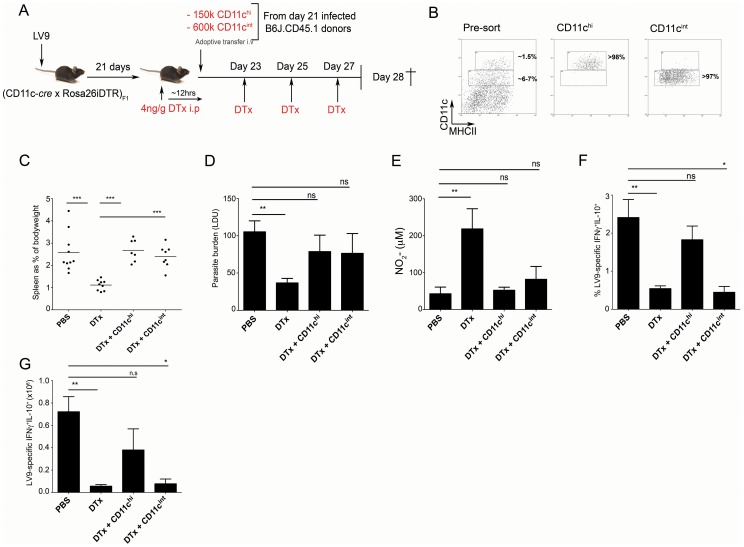
CD11c^hi^ cDCs are required for the expansion/maintenance of IFNγ^+^IL-10^+^ CD4^+^ T cells. (**A**) 12 h after initiation of DTx treatment, (CD11c-*cre*×Rosa26iDTR)_F1_ mice were reconstituted i.v. with CD11c^hi^MHCII^hi^ cDCs or CD11c^lo/int^ cells obtained from day 21-infected congenic B6J.CD45.1 donors. (**B**) Purity of transferred cells. (**C**–**E**) Splenomegaly (C), spleen parasite burden (D), and spontaneous NO production (E) in recipient mice. (**F and G**) The frequency (F) and number (G) of antigen-specific CD4^+^ T cells producing IFNγ and IL-10 in recipient mice. Data are pooled from two independent experiments (n = 7–10 mice). * = p<0.05, ** = p<0.01, *** = p<0.001 for indicated comparisons between groups.

As neutrophils play a role in the control of established *L. donovani* infection [45] and splenic neutrophil numbers increase after DTx treatment (this study and [46], [47]), we repeated these experiments using DTx-treated infected mice co-treated with a Ly6G-specific mAB (1A8) to deplete neutrophils. The frequency of IFNγ^+^IL-10^+^ CD4^+^ T cells was similar in DTx-treated mice irrespective of whether neutrophils were present or absent (1.69±0.37% vs. 1.89±0.40% in control and 1A8-treated mice respectively). Neutrophil depleted DTx-treated mice also showed a similar reduction in splenomegaly and increased NO production as seen in neutrophil replete DTx-treated mice (**Figure 8A and B**). As previously noted in wild type mice [45], neutrophil depletion of DTx-treated mice led to increased parasite burden (**Figure 9C**), illustrating that immunopathology is not strictly associated with parasite load. Nevertheless, changes in neutrophil number cannot account for the changes in Th1 cell differentiation or immunopathology observed in DTx-treated mice. Hence, these data demonstrate that depletion of CD11c^+^ cells during ongoing infection dramatically reduces splenic pathology, promotes NO-dependent parasite clearance and significantly impairs the generation of antigen-specific CD4^+^ T cells capable of simultaneous IL-10 and IFNγ production, whilst only slightly reducing the abundance of other Th subsets.

### CD11c^hi^ cDCs are required for the expansion/maintenance of IFNγ^+^IL-10^+^ CD4^+^ T cells

To definitively address whether cDCs or other populations of CD11c^+^ cells were responsible for the induction of IL-10 production in Th1 cells and for changes to host resistance, we employed a functional complementation approach. We adoptively transferred (in accordance with their population abundance) wild type DTx-resistant CD11c^hi^MHCII^hi^ cDCs and CD11c^int/lo^ cells from infected B6.CD45.1 mice into infected DTx-treated (CD11c-*cre*×Rosa26iDTR)_F1_ mice (**Figure 9A and B**). cDCs obtained at d21 p.i. had similar patterns of co-stimulatory molecule expression to cDCs obtained at d28 p.i., particularly with regard to PD-L1 expression and this was also similar to the phenotype of CD11c^int^ cells (in spite of heterogeneity within this population; **Figure S5**). We also characterised CD11c^int^ cells and cDCs for IL-27p28 (**Figure S6A**) and IL-10 (**Figure S6B**) mRNA accumulation at d21 p.i. and day 28 p.i. and observed significantly lower accumulation of both cytokines within the CD11c^int^ population, suggesting a sustained difference in their capacity to regulate IL-10 and IL-27 expression during infection. Ablation of endogenous CD11c^+^ cells was maintained after transfer by repeated DTx treatment. Strikingly, transfer of either CD11c^int/lo^ cells or CD11c^hi^ cDCs was sufficient to restore splenomegaly (**Figure 9C**), parasite burden (**Figure 9D**) and NO production (**Figure 9E**) to levels similar to that observed in non-treated infected mice. The potency of these cells to restore disease progression was all the more remarkable given that at the time of assay (d7 post transfer) donor CD45.1 CD11c^+^ cells could not be detected, suggesting long term engraftment had not occurred (data not shown). Adoptive transfer had no impact on the frequency of IFNγ single-producing CD4^+^ T cells (in keeping with the limited effect of CD11c depletion on this T cell response) or on the frequency of IL-10^+^ single producing T cells, although absolute numbers were increased as a result of the changes in splenomegaly after these interventions (not shown). In contrast, adoptive transfer of cDCs, but not CD11c^int/lo^ cells, restored the frequency (**Figure 9F**) and absolute numbers (**Figure 9G**) of IFNγ^+^IL-10^+^ CD4^+^ T cells to that observed in untreated infected mice. cDCs are therefore required to promote *in vivo* expansion of IFNγ^+^IL-10^+^ CD4^+^ T cells during *L. donovani* infection.

## Discussion

This study is the first to demonstrate that CD11c^+^ cells act to promote disease progression during the chronic phase of infection with *L. donovani*. Furthermore, by combining conditional DTx-mediated depletion with adoptive transfer during ongoing infection, we could show that whereas both CD11c^hi^ and CD11c^int/lo^ cells contribute to disease progression and suppress host protective immunity, only CD11c^hi^ cells are capable of promoting the expansion and/or maintenance of Th1 cells that produce IL-10. In addition to providing the first evidence that cDCs are required to promote expansion of CD4^+^ T cells with mixed effector/regulatory phenotype *in vivo*, our data suggests that the emergence of Th1 cells producing IL-10 is not essential for disease progression.

Unlike previous studies assessing the role of DCs in acute *L. donovani* infection [48] or Langerhans cells in acute *L. major* infection [15], sustained ablation of CD11c-expressing cells during ongoing infection was required in this study. Under the conditions used, we could deplete 80–90% of splenic CD11c^hi^MHCII^hi^ cells in mice chronically infected with *L. donovani*, similar to the efficacy reported for DC depletion during *Schistosoma mansoni* infection [49]. The impact of CD11c^+^ cell ablation on disease progression was striking, with reduced splenic pathology, enhanced nitric oxide (NO) production and enhanced parasite clearance. Indeed, the impact of CD11c depletion was of a similar magnitude to that observed after a variety of chemotherapeutic and immunotherapeutic interventions [50]. Neutrophil influx has recently been noted following DTx treatment of mice [46], [47] and was also observed by us here. It is unclear whether CXCL2-mediated egress from the bone marrow underlies the neutrophilia in (CD11c-*cre*×Rosa26iDTR)_F1_ mice, as suggested for other strains [47]. In preliminary studies, we have also noted a significant increase in the abundance for *Il17α* mRNA after DTx treatment (data not shown), but further studies are required to determine whether this cytokine also impacts on neutrophil recruitment. In spite of this influx of phagocytes, simultaneous depletion of neutrophils *in vivo* demonstrated that even in their absence, the ablation of CD11c^+^ cells resulted in a marked reduction in splenomegaly and increased levels of NO production. Of note, parasite burden was increased in neutrophil depleted DTx treated mice, even though NO levels also increased, suggesting that the leishmanicidal effect of neutrophils may be mediated through NO-independent mechanisms. Nevertheless, to more directly overcome the pitfalls associated with off-target effects of DTx treatment, and to more formally address the role of cDCs in disease progression, we employed a strategy that allowed simultaneous depletion of endogenous CD11c^+^ cells and reconstitution with subpopulations of wild type CD11c^+^ cells. Similar to a recent study of allergic inflammation [51], the transfer of relatively small numbers of highly purified CD11c^hi^ cDCs to CD11c-ablated mice resulted in substantial modulation of disease. All parameters of host resistance that we measured were suppressed after CD11c^+^ cell transfer and splenomegaly returned to the level observed in untreated infected mice. Importantly, we could not distinguish between the effects of transfer of CD11c^hi^ cDCs and CD11c^int/lo^ cells based on these criteria.

Splenic cDCs capable of promoting disease progression had a number of characteristics that distinguish them from cDCs found in naïve mice, with only minor differences seen between subsets. CD80 and CD86 expression is muted during chronic infection, similar to what has been observed at early times (∼5 hr) post infection [18], [52]. CD40 expression was somewhat higher at day 28 p.i. than at d21 p.i., but there are conflicting reports as to whether the CD40-CD40L axis is required [53], [54] or redundant [55], [56] with respect to anti-*Leishmania* responses. It has been suggested that signaling downstream of CD40 may perpetuate IL-10 production and enhance productive infection of macrophages [57] but this has not been evaluated in DCs. cDCs from chronically infected mice showed enhanced expression of Programmed Death Ligand 1 (PD-L1), a negative costimulatory molecule involved in regulating functional exhaustion of CD8^+^ T cells during *L. donovani* infection [58]. IL-12p70 production was severely impaired, whereas high levels of spontaneous IL-10 and IL-27 were observed, with some differences between subsets also reflected in previous data at the mRNA level [18]. IL-10 produced by cDCs in infected mice appears to play an important autocrine regulatory role in limiting IL-12p70 production. Previous work has identified splenic cDCs as being particularly sensitive to autocrine regulation of IL-12 production by IL-10 *in vitro* [42] and although the molecular mechanisms for such a process are not fully described, Stat3 has been shown to mediate some of the inhibitory effects of IL-10 on cDC activation *in vivo* [59]. In contrast, we found no evidence supporting a role for IL-27 in the regulation of IL-12p70 or IL-10 production in cDCs, despite evidence that BMDCs generated from IL-27Rα^−/−^ mice show enhanced production of IL-12p40 and p70 in response to TLR ligation [60] and that autocrine IL-27 is required for optimal macrophage IL-10 production [61].

The CD11c^int/lo^ population capable of transferring disease progression includes NK cells and CD11c^lo^CD45RB^+^ ‘regulatory’ DCs, two populations that we have previously shown to produce IL-10 and contribute to immunopathology using other assay systems [21], [43]. These data are also consistent with evidence that IL-10 production by innate cells, rather than T cells, is the dominant negative regulator of effector responses after vaccination against *L. major* [62], and that therapeutic infusion of LPS-activated BMDCs reduces pathology and parasite load, irrespective of whether the splenic IL-10-producing CD4^+^ T cell frequency is reduced or maintained [23]. IL-10 is a well known suppressor of IFNγ-induced NO production [63]. Hence, the loss of IL-10 expressing cDCs and CD11c^int^ cells in an IFNγ-replete environment may underlie the increased NO production observed after therapeutic depletion of CD11c^+^ cells.

Unlike previous data showing a requirement for myeloid DCs in the generation of effector responses to acute *L. donovani* infection [48], the ablation of CD11c^+^ cells during chronic infection did not significantly affect IFNγ production by CD4^+^ T cells, suggesting that neither cDCs nor other CD11c^+^ cells are essential for the maintenance of effector T cell responses, at least over the 7 day time frame studied here. This result is in keeping with data showing the effects of DC ablation on CD4^+^ T cell responses to *M. tuberculosis*, where DCs are critical for initial priming of CD4^+^ effector T cell responses but dispensable for recall Th1 responses after vaccination [6]. However our data are in contrast to a report showing that CD11c-depletion during established infection with *S. mansoni* resulted in a significant reduction in IFNγ^+^ production by CD4^+^ T cells after ablation of CD11c-expressing cells [49]. Hence, the requirement for CD11c^+^ cells to maintain T cell IFNγ production would appear to be context and/or infection-specific.

Chronic *L. donovani* infection is associated with the expansion of CD4^+^ T cells that co-express both IFNγ and IL-10 (this manuscript and [23], [24]). The further detailed characterization of this population provided here indicates that during *L. donovani* infection, these IL-10-producing CD4^+^ IFNγ^+^ T cells are Foxp3^−^, T-bet^+^ and CD127^−^. Hence, they are likely to be related to the IL-10-producing effector T cells found in experimental *Toxoplasma gondii* and *Listeria monocytogenes* infection [35], [64] and *Plasmodium* infection [31], [65]. Although T-bet and CD127 expression were not directly addressed, Foxp3^−^ ‘effector’ CD4^+^ T cells also appear to be the major CD4^+^ T cell subset producing IL-10 during infection of mice with *L. major* [66], [67]. However the increase in Foxp3^+^ natural Treg frequency and suppressive activity seen in cutaneous *L. major* infection [68] appears to be absent during EVL (this manuscript and [23]). IFNγ^+^IL-10^+^ cells are also associated with *L. donovani* and *Mycobacterium tuberculosis* infection in humans [69], [70].

Multiple lines of evidence have suggested a link between IL-27 and the production of IL-10 by CD4^+^ T cells *in vitro* or during autoimmune processes [27], [28], [29], [30], [32], [33]. IL-27 plays a role in the development of IFNγ^+^IL-10^+^ CD4^+^ T cells during experimental *L. major* [71] and *Listeria monocytogenes* [35] infection *in vivo*, as assessed using IL-27Rα^−/−^ mice. Such mice also display enhanced resistance to infection with *L. donovani*, although T cell IL-10 production was not assessed [72]. Furthermore, systemic IL-27 levels are elevated in humans infected with *L. donovani*, with splenic myeloid cells providing a major source of IL-27 mRNA that was proposed to enhance IFNγ^+^IL-10^+^ T cell responses via the induction of T cell-derived IL-21 [73]. Although there is some *in vitro* evidence of DC-derived IL-27 inducing T cell IL-10 production [37], [38], none of the studies described above provided a formal and causal link between IL-27-producing cDCs and IFNγ^+^IL-10^+^ CD4^+^ cell polarization *in vivo*, as we have now demonstrated here. The capacity for cDCs to drive polarization of IFNγ^+^IL-10^+^ cells may be as a result of their higher expression of IL-27 than CD11c^int^ cells during infection. However, we believe it is likely that a combination of cytokine profile and the capacity for sustained antigen presentation underlies the essential requirement for CD11c^hi^ cDCs in the generation of IFNγ and IL-10 co-producing CD4^+^ T cells [64], [74], rather than sole production of IL-27. Further study and the development of mice with targeted deficiency of IL-27p28 will be required to delineate the relative contribution of these events *in vivo*.

Although T cell-derived IFNγ has long been known as a critical mediator of parasite clearance in EVL [75], the role of Th1 cells producing mixed effector/regulatory cytokines still remains to be clearly established. During infections where pro-inflammatory responses would otherwise be rampant, this phenotype appears essential to minimize host-mediated pathology [31], [64]. However, the association of IFNγ^+^IL-10^+^ CD4^+^ T cells with disease progression in leishmaniasis has suggested that through IL-10 production, these cells may contribute to parasite persistence and/or disease pathology. The data provided in this manuscript suggest that at least in EVL this association is not causal, as splenomegaly and loss of host resistance were equally well promoted by CD11c^int/lo^ cells as by CD11c^hi^ cells, even though the former failed to promote the expansion/maintenance of IL-10-producing Th1 cells. A similar conclusion was also drawn from reciprocal studies using a model of DC immunotherapy [23]. Our data indirectly suggest, therefore, that IL-10 derived from other cellular sources is sufficient to counterbalance the otherwise potentially fatal consequences of Th1-derived effector cytokines.

On a cautionary note, whilst it is tempting to conclude that there is a causal link between the phenotype of cDCs (and CD11c^int^ cells), the regulation of T cell immunity, parasite containment and the development of pathology, this may not be the case, given the complexity of potential interactions *in vivo*. For example, we have shown that cDCs from infected mice retain the capacity for antigen presentation *in vitro* (data not shown) and altered activation of T cells is clearly a consequence of cDC transfer. Studies involving the transfer of MHC-deficient cDCs (and CD11c^int^ cells) would be required, however, to discover whether cDCs and CD11c^int^ cells also have the potential to regulate pathology independently of their capacity to interact in a cognate manner with T cells, e.g. by influencing local stromal or myeloid cell function or directly regulating vascular remodeling [43], [45], [50].

Finally, the recognition that cDCs switch from a host protective role in the induction of immunity [48] to one in which they may hinder parasite elimination may provide exciting new opportunities for targeted immunotherapy. Indeed, the impact of CD11c^+^ cell depletion was of a similar magnitude to that observed after a variety of chemotherapeutic and immunotherapeutic interventions (reviewed in [76]). To our knowledge, DC depletion with the aim of overcoming immunosuppression has not been attempted in the clinic, though removing excessively stimulatory DCs has been suggested as a therapeutic approach to prevent GVHD after allogeneic hematopoietic stem cell transplantation [77]. Whether DCs would serve as potential targets for short-term antibody-based immunotherapy in human VL remains to be determined and will require further concerted efforts to first characterize human DC subsets and their function during this disease.

## Materials and Methods

### Ethics statement

All animal care and experimental procedures were carried out after review and approval by the University of York Ethical Review Process, and conducted under the authority of United Kingdom Home Office Project Licence PPL 60/3708 (‘Immunology and Immunopathology and visceral leishmaniasis’). All experiments were designed and conducted to minimise suffering and to comply with the principles of replacement, refinement and reduction.

### Mice and infections

C57BL/6 and B6J.CD45.1 mice were obtained from the Biological Services Facility (University of York) or supplied by Charles River Laboratories. C57BL/6J-Tg (Itgax-*cre*-EGFP) 4097Ach/J (CD11c-*cre*) mice and C57BL/6-*Gt(ROSA)26Sor^tm1(HBEGF)Awai^*/J (Rosa26iDTR) mice were obtained from The Jackson Laboratory (Bar Harbor, Maine, USA) and *Cre* and eGFP genotype positive F1 mice were used between 6 and 12 weeks of age. Mice were infected via the lateral tail vein with 3×10^7^ amastigotes of the Ethiopian strain of *Leishmania donovani* (LV9). Splenomegaly was calculated relative to body weight and parasite burdens were quantified as Leishman-Donovan Units (LDU) [23]. (CD11c-*cre*×Rosa26iDTR)_F1_ mice were treated with 4 ng/g Diphtheria toxin from *Corynebacterium diptheriae* (DTx, Sigma) i.p. and/or treated with mAb 1A8 or control [45] every other day from day 21 of infection, as required. Administration of 1A8 resulted in depletion of ∼90% of splenic neutrophils, as judged by CD11b, Ly6C and Gr-1 staining (data not shown).

### Flow cytometry

Spleen cells were restimulated for either 90–120 min with 10 ng/ml PMA and 1 µg/ml Ionomycin (Sigma-Aldrich, UK) or for 3 h with BMDCs pulsed with fixed *L. donovani* amastigotes, and then further incubated with 1 µg/ml Brefeldin A for 4 h. After restimulation, cells were labeled for 30 minutes on ice with mAbs: CD3ε-PE-Cy7 (145-2C11), CD4-FITC (RM4-5), CD127-PE (A7R34) (eBioscience) or CD4-PerCP (RM4-5; BD Pharmingen). Cells were washed and incubated for 30 min on ice in PBS containing Fixable Viability Dye eFluor^780^ (eBioscience). After washing, cells were fixed (15 min on ice) in 2% paraformaldehyde (PFA). Cells were then permeabilised using 1% Saponin (Sigma PERM buffer). Cells were subsequently labeled (45–60 min on ice) in PERM buffer containing mAbs: IFNγ-PacificBlue (XMG1.2), IFNγ-eFluor^450^ (XMG1.2), IL-10-APC (JES5-16E3), IL-10-PE (JES5-16E3) T-bet-AlexaFluor^647^ (ebio4BIO) and Foxp3-FITC (FJK-16a), (eBioscience). All cells were analyzed on a CyAN-ADP flow cytometer using Summit Software (Beckman Coulter, USA).

### Antigen-specific restimulation of CD4^+^ T cells *in vitro*

BMDCs were generated from femurs of C57BL/6 mice using standard methods. On day 7 of culture, cells were pulsed for 24 hrs with paraformaldehyde-fixed *Leishmania donovani* amastigotes at a ratio of 100 amastigotes to 1 BMDC. Antigen-pulsed BMDCs were subsequently used to restimulate T cells for 3 hours, prior to addition of Brefeldin A for 4 hours and subsequent assessment of CD4^+^ T cell cytokine production by intracellular flow cytometric analysis, as previously described.

### Dendritic cell subset isolation and staining

Spleens were dissociated mechanically and digested in 0.2 mg/ml collagenase type IV/DNAse1 mix (Worthington Biochemical, NJ, USA) for 30 minutes at room temperature. Staining was performed as above using mAbs: CD11c-PE-Cy7 (N418), Major Histocompatibility complex class II (MHCII)-APC (M5/114.15.2), MHCII-eFluor^450^ (M5/114.15.2), CD8α-FITC (53-6.7), CD4-APC (RM4-5), CD40-PE (1C10), CD80-PE (16-10A1), CD86-PE (GL1) and B7-H1-PE (MIH5). For purification, CD11c^+^ cells were enriched by magnetic separation [18] and CD11c^hi^MHCII^hi^ cDCs or individual cDC subsets were sorted to ∼98–99% purity on a BeckmanCoulter MoFlo cell sorter.

### cDC cytokine production determined by ELISA

After sorting, cells were washed, counted and plated in triplicate in complete RPMI at 1×10^6^ cells/ml. Where indicated, LPS (1 µg/ml; Sigma), anti-mouse IL-27p28 or Goat IgG (10 µg/ml; R&D Systems) or anti-mouse IL-10R (1.3 µg/ml; gift of Dr. M. Kullberg) were added. After 24 h, supernatants were harvested and stored at −80°C until assessed by ELISA for levels of IL-12p40, IL-10 (Mabtech, Sweden), IL-27p28 and IL-12p70 (R&D Systems).

### Nitric oxide production

Splenocytes from naïve and infected PBS or DTx-treated mice were incubated in RPMI at 5×10^6^ cells/ml for 60 min at 37°C, and non-adherent cells removed by vigorous washing. Adherent cells were cultured for 24 h at 37°C and supernatants were assayed using Greiss Reagent (Promega, Madison, WI, USA).

### Adoptive transfer

12 h after the first administration of DTx, infected (CD11c-*cre*×Rosa26iDTR)_F1_ mice received 1.5×10^5^ CD11c^hi^MHCII^hi^ cells or 6.0×10^5^ CD11c^int^MHCII^+^ cells isolated from day 21-infected B6J.CD45.1 mice. DTx administration was continued at 48 h intervals to maintain depletion of endogenous CD11c^+^ cells.

### Statistical analysis

Statistical analysis was performed using a students t test or one-way ANOVA as appropriate, with p<0.05 considered significant. All experiments were conducted independently at least twice.

## Supporting Information

Figure S1 **CD4^+^Foxp3^+^ T cells do not increase in frequency during chronic infection.** The frequency of splenic CD4^+^ Foxp3^+^ natural Tregs was determined by intracellular flow cytometry in naïve and day 28-infected mice. Representative flow plots (A) shown alongside chart of mean frequency (±SEM) of Foxp3^+^ CD4 T cells from n = 3 mice per group (B). Representative of 2 experiments. * = p<0.05 for infected vs naïve mice.
(TIF)

Figure S2 **Costimulatory molecule expression on cDCs during chronic infection.** Splenic CD11c^hi^MHCII^hi^ cDCs from naïve (filled histogram) and day 28-infected (open histogram) mice were assessed by flow cytometry for surface expression of the indicated costimulatory molecules. Representative histograms for experimental groups referred to in Figure 2.
(TIF)

Figure S3 ***Tgfβ1*** **and** ***Ido*** **mRNA accumulation in cDCs from naïve and infected mice.** (**A–B**) mRNA accumulation for *Tgfβ1* (A) and *Ido* (B) was determined by quantitative RT-PCR in cDC subsets isolated from naïve and day 28 infected mice. Representative of 2–3 independent experiments (n = 4 mice per group). ** = p<0.01.
(TIF)

Figure S4 **Alterations in splenic immune cell composition after DTx administration to (CD11c-** ***cre*** **×Rosa26iDTR)_F1_ mice.** Alterations in the frequency and number of splenic T and B cells (A & B ), CD4+ and CD8**α**+ T cells (C &D ), NK cells (E & F ) and neutrophils (G & H ) were assessed by flow cytometry after administration of PBS (open bars) or DTx (closed bars) to (CD11c-*cre*×Rosa26iDTR)_F1_ mice over a 7 day period. Data are mean ± SEM from 4–5 mice per group and representative of two or three separate experiments. * = p<0.05 for DTx versus PBS treated mice.
(TIF)

Figure S5 **Co-stimulatory molecule expression by splenic CD11c^+^ cells at day 21 post infection.** Surface expression of the indicated co-stimulatory molecules was assessed on splenic CD11c^hi^MHCII^hi^ cDCs and CD11c^int^ cells from naïve and day 21-infected mice by flow cytometry. Flow plots and histograms are representative, Grey filled indicates isotype control, Blue open lines indicate cells from naïve mice and Red open lines indicate cells from mice at day 21 p.i. Charts show mean fold change in surface expression of indicated molecule on indicated subset ±SEM compared to naïve mice n = 5.
(TIF)

Figure S6 **Differential accumulation of** ***Il10*** **and** ***Il27p28*** **mRNA by CD11c^+^ cells during infection.** CD11c^hi^MHCII^hi^ cDCs and CD11c^int^ cells were sorted from spleens of individual naïve, day 21 and day 28 infected mice. Levels of *Il10* and *Il27p28* were assessed in sorted populations by qRT-PCR. **A** and **B** show mean fold change (±SEM) in the levels of *Il10* and *Il27p28* mRNA in indicated individually sorted cell populations at the time point indicated, relative to the mean levels of *Il10* or *Il27p28* in the relevant subset sorted from n = 3 individual naïve mice, assessed using *Hprt* as an endogenous control. Cells were sorted from individual n = 3 naïve and n = 5 day 21 and day 28-infected mice. * = p<0.05, *** = p<0.001.
(TIF)
