# Supplementary figures and images for: IL-10-Producing Th1 Cells and Disease Progression Are Regulated by Distinct CD11c+ Cell Populations during Visceral Leishmaniasis
Source: PLoS Pathog. 2012 Jul 26;8(7):e1002827. doi: 10.1371/journal.ppat.1002827 (PMC3406093; doi:10.1371/journal.ppat.1002827)

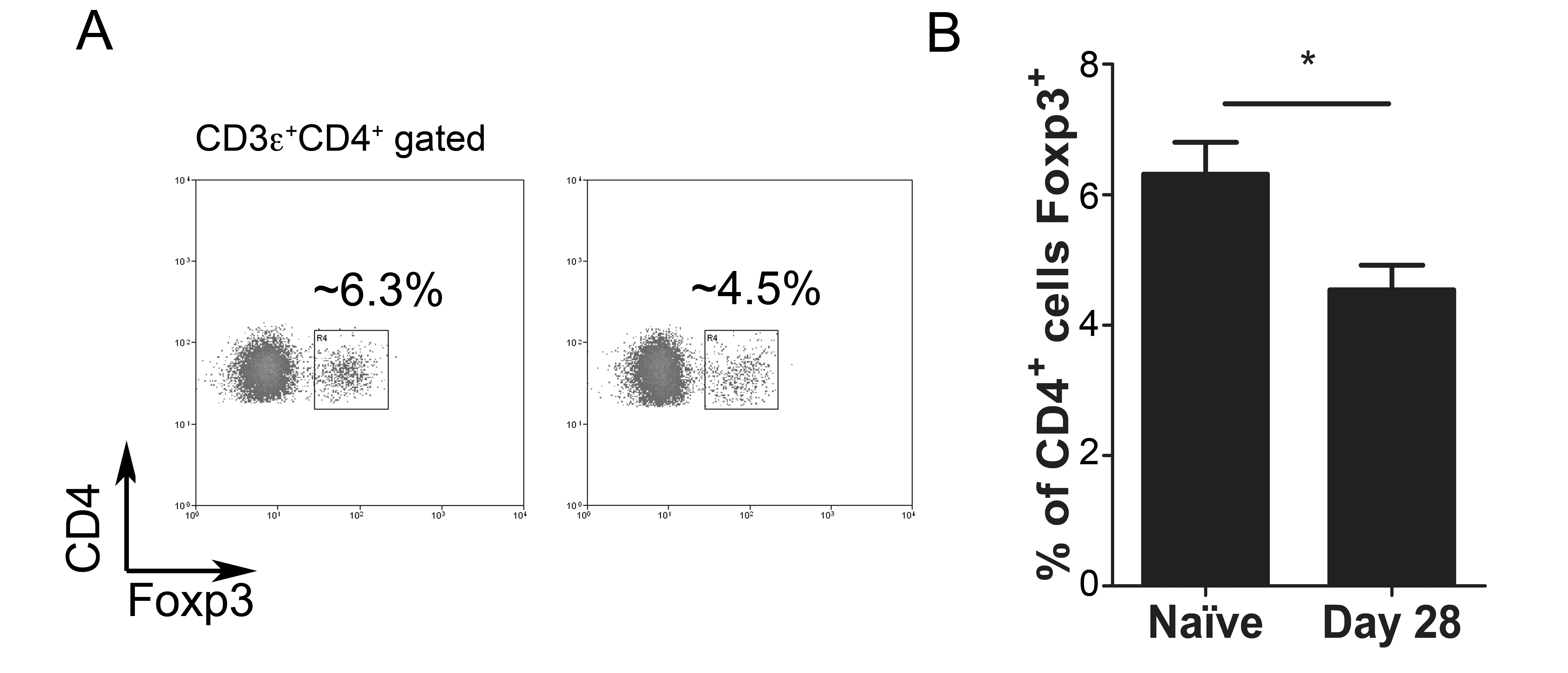

Supplement: Figure S1 — CD4+Foxp3+ T cells do not increase in frequency during chronic infection. The frequency of splenic CD4+ Foxp3+ natural Tregs was determined by intracellular flow cytometry in naïve and day 28-infected mice. Representative flow plots (A) shown alongside chart of mean frequency (±SEM) of Foxp3+ CD4 T cells from n = 3 mice per group (B). Representative of 2 experiments. * = p<0.05 for infected vs naïve mice. (TIF) [file ppat.1002827.s001.tif]

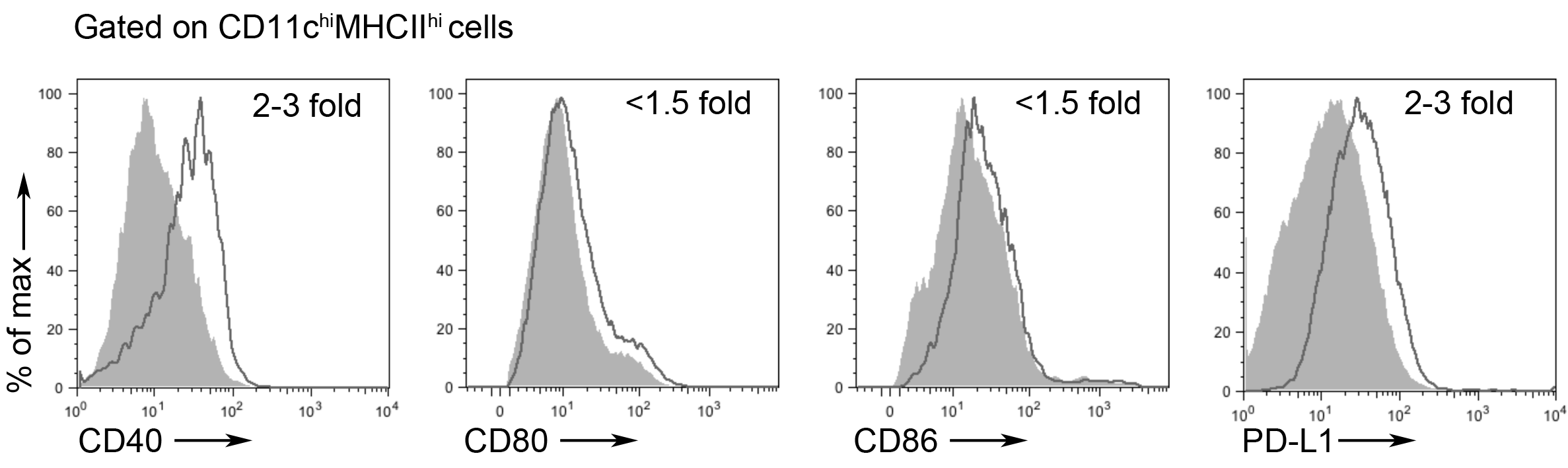

Supplement: Figure S2 — Costimulatory molecule expression on cDCs during chronic infection. Splenic CD11chiMHCIIhi cDCs from naïve (filled histogram) and day 28-infected (open histogram) mice were assessed by flow cytometry for surface expression of the indicated costimulatory molecules. Representative histograms for experimental groups referred to in Figure 2. (TIF) [file ppat.1002827.s002.tif]

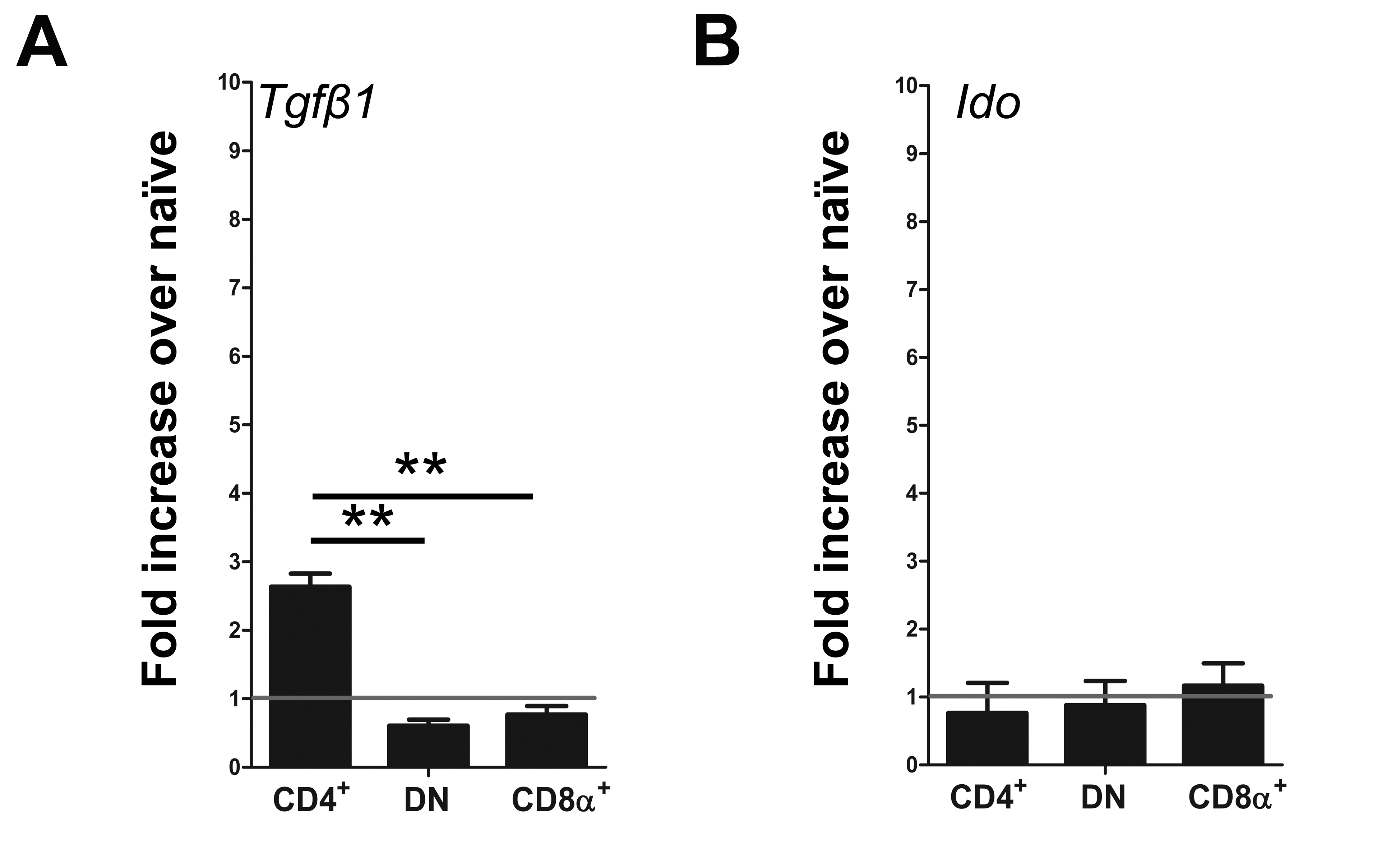

Supplement: Figure S3 — Tgfβ1 and Ido mRNA accumulation in cDCs from naïve and infected mice. (A–B) mRNA accumulation for Tgfβ1 (A) and Ido (B) was determined by quantitative RT-PCR in cDC subsets isolated from naïve and day 28 infected mice. Representative of 2–3 independent experiments (n = 4 mice per group). ** = p<0.01. (TIF) [file ppat.1002827.s003.tif]

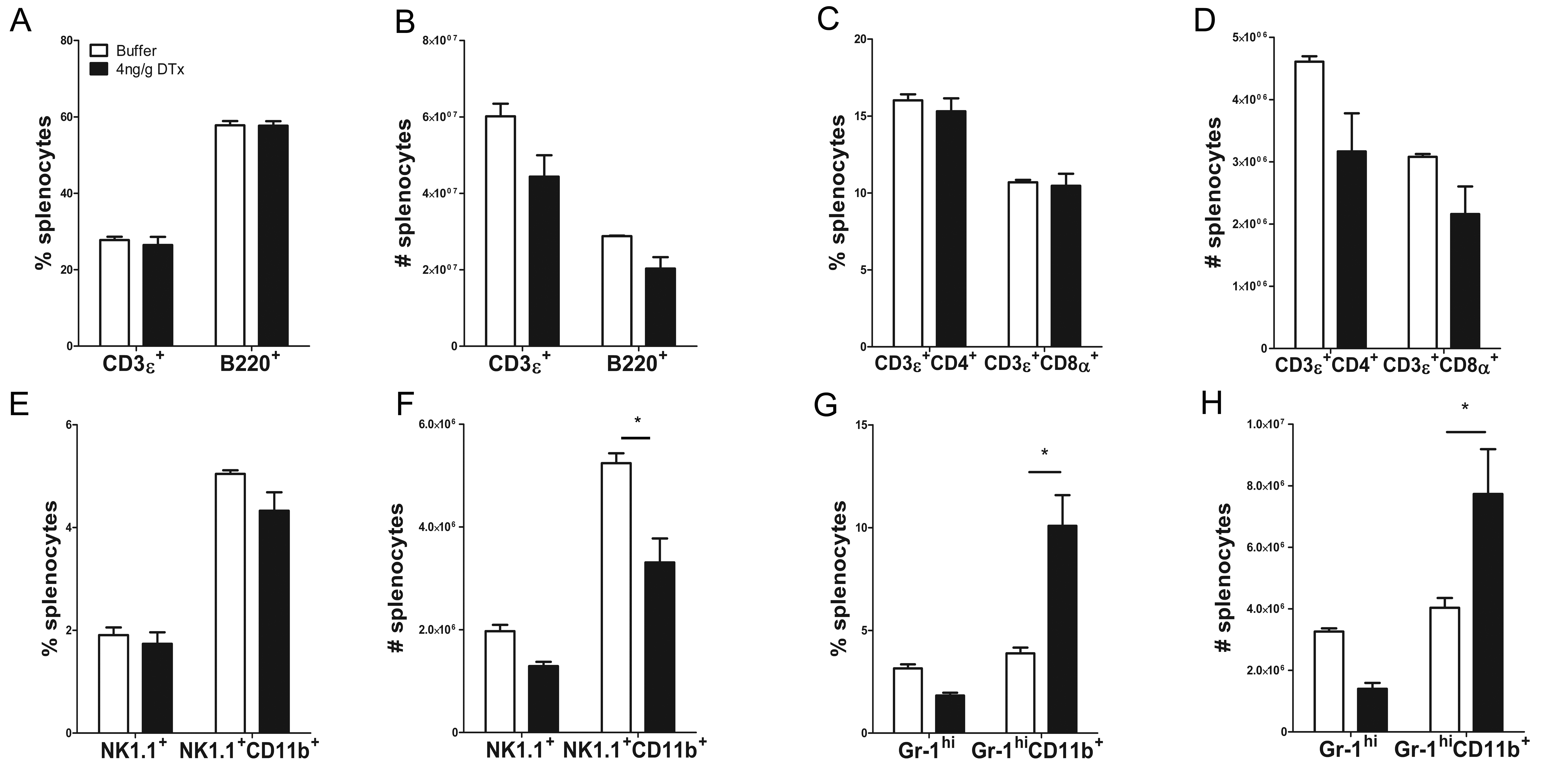

Supplement: Figure S4 — Alterations in splenic immune cell composition after DTx administration to (CD11c- cre ×Rosa26iDTR)F1 mice. Alterations in the frequency and number of splenic T and B cells (A & B ), CD4+ and CD8α+ T cells (C &D ), NK cells (E & F ) and neutrophils (G & H ) were assessed by flow cytometry after administration of PBS (open bars) or DTx (closed bars) to (CD11c-cre×Rosa26iDTR)F1 mice over a 7 day period. Data are mean ± SEM from 4–5 mice per group and representative of two or three separate experiments. * = p<0.05 for DTx versus PBS treated mice. (TIF) [file ppat.1002827.s004.tif]

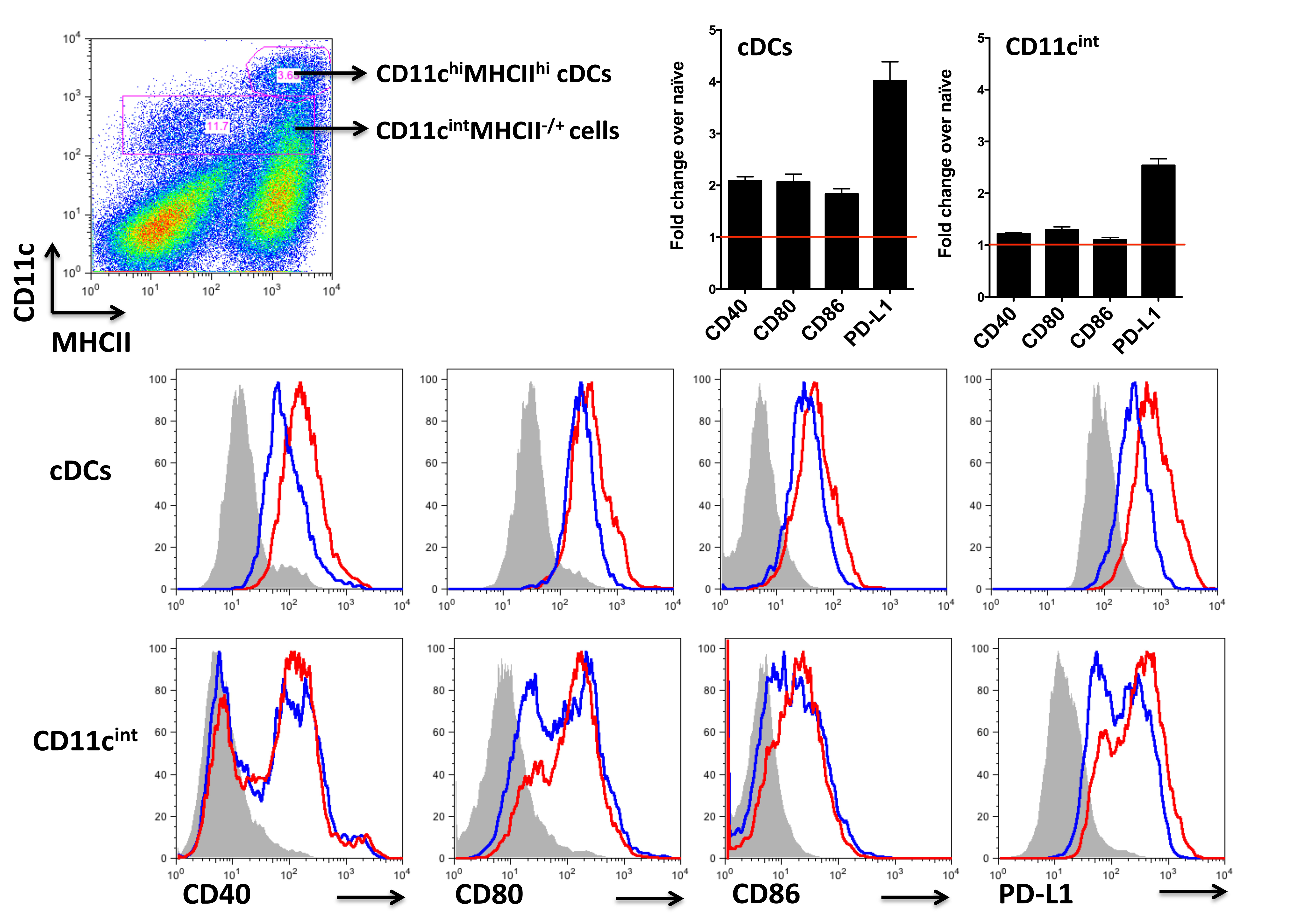

Supplement: Figure S5 — Co-stimulatory molecule expression by splenic CD11c+ cells at day 21 post infection. Surface expression of the indicated co-stimulatory molecules was assessed on splenic CD11chiMHCIIhi cDCs and CD11cint cells from naïve and day 21-infected mice by flow cytometry. Flow plots and histograms are representative, Grey filled indicates isotype control, Blue open lines indicate cells from naïve mice and Red open lines indicate cells from mice at day 21 p.i. Charts show mean fold change in surface expression of indicated molecule on indicated subset ±SEM compared to naïve mice n = 5. (TIF) [file ppat.1002827.s005.tif]

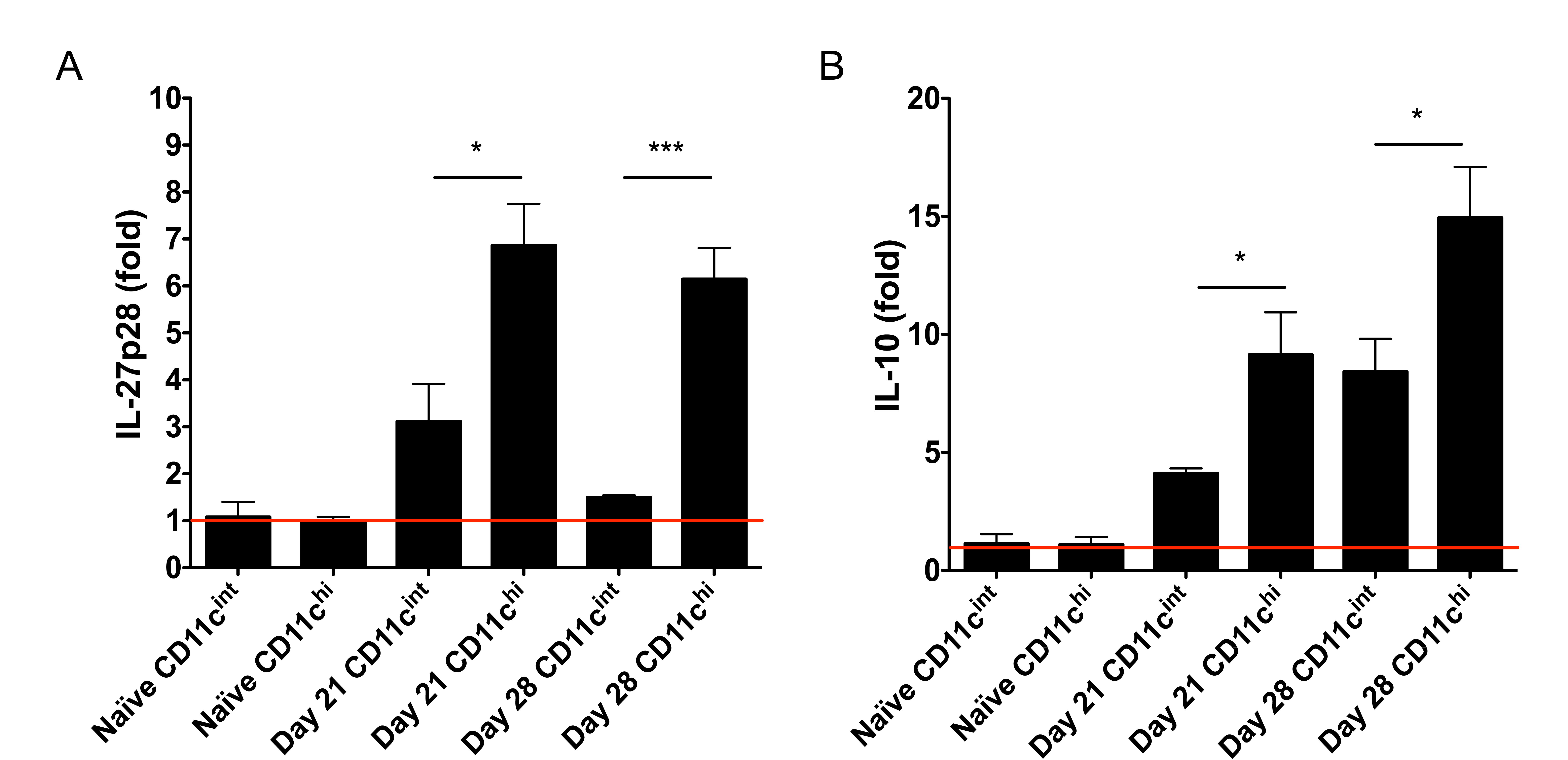

Supplement: Figure S6 — Differential accumulation of Il10 and Il27p28 mRNA by CD11c+ cells during infection. CD11chiMHCIIhi cDCs and CD11cint cells were sorted from spleens of individual naïve, day 21 and day 28 infected mice. Levels of Il10 and Il27p28 were assessed in sorted populations by qRT-PCR. A and B show mean fold change (±SEM) in the levels of Il10 and Il27p28 mRNA in indicated individually sorted cell populations at the time point indicated, relative to the mean levels of Il10 or Il27p28 in the relevant subset sorted from n = 3 individual naïve mice, assessed using Hprt as an endogenous control. Cells were sorted from individual n = 3 naïve and n = 5 day 21 and day 28-infected mice. * = p<0.05, *** = p<0.001. (TIF) [file ppat.1002827.s006.tif]
